# Simultaneous Percutaneous Ventricular Septal Closure and Mitral Valve Repair in Postinfarction Mechanical Complications: Case Report

**DOI:** 10.1016/j.jscai.2025.103825

**Published:** 2025-08-21

**Authors:** Azka Naeem, Ilya Giverts, Muhammad Hashim Khan, Aakash Shetty, Vijay Shetty, Benjamin Youdelman, Robert Frankel, Paul Saunders, Habib-Hymie Chera

**Affiliations:** a Department of Internal Medicine, Maimonides Medical Center, Brooklyn, New York; b Department of Oncology, Rutgers, the State University of New Jersey, New Brunswick, New Jersey; c Division of Cardiothoracic Surgery, Maimonides Medical Center, Brooklyn, New York; d Department of Cardiovascular Medicine, Maimonides Medical Center, Brooklyn, New York

**Keywords:** acute myocardial infarction, amplatzer device, cardiogenic shock, case report, mitral regurgitation, papillary muscle rupture, transcatheter edge-to-edge mitral valve repair

## Abstract

Ventricular septal rupture (VSR) and papillary muscle rupture are lethal complications of acute myocardial infarction. We present the case of a 76-year-old woman presenting with chest pain with electrocardiogram showing ST-segment elevation in the inferior leads. Angiography showed chronic right coronary artery occlusion. A VSR and partially torn papillary muscle was seen on echocardiogram. Percutaneous VSR closure with an Amplatzer device (Abbott) and mitral valve repair with a MitraClip (Abbott) were successfully performed simultaneously, and she was discharged to a skilled nursing facility. Percutaneous intervention seems a promising alternative, even for VSR and papillary muscle rupture in a single setting.

## Introduction

The incidence of mechanical complications of acute myocardial infarction (AMI) has decreased since the implementation of early reperfusion strategy. However, hospital mortality remains high, particularly in older patients, and is reported to be between 10% and 40%.^1^^,^^2^ Short-term mechanical complications after AMI include acute mitral regurgitation (MR) secondary to papillary muscle rupture (PMR), ventricular septal rupture (VSR), and ventricular free wall rupture. PMR causes up to 7% of patients to have cardiogenic shock after myocardial infarction (MI) and is usually diagnosed after 2 to 7 days. VSR also typically occurs after 3 to 5 days, with an approximate incidence of 0.21% of ST-elevation MI (STEMI) cases and 0.04% of non-STEMI cases. First MI, STEMI, older age, prior stroke, female sex, elevated cardiac markers, higher heart rate, lower blood pressure, higher Killip class, and delayed or absent reperfusion are all associated with an increased risk of developing postinfarct VSR.^3^, ^4^, ^5^ While surgery remains the only definitive treatment, it is associated with a high-risk of mortality and morbidity, ranging up to 54.1% in first week.^6^ The timing of surgery remains a subject of debate; conservative treatment leads to a 30-day mortality rate exceeding 90%, whereas urgent surgical repair of post-MI VSR in patients with cardiogenic shock and respiratory failure is also associated with a mortality rate of 40%.^7^ Hence, percutaneous closure is emerging as a treatment option for patients with prohibitive surgical risks.^8^^,^^9^ Hence, we present a case of successful correction of post-MI VSR and PMR by simultaneous percutaneous intervention simultaneously.

## Case presentation

**Figure 1:**
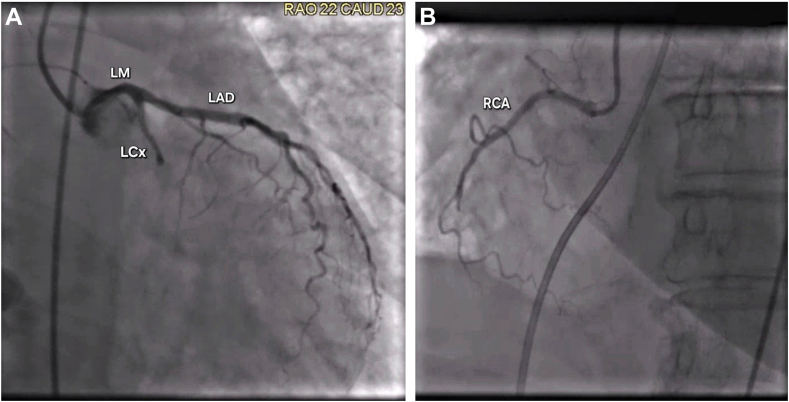
**Left heart catheterization.** (**A**) Right anterior oblique caudal view showing left anterior descending artery (LAD) and 100% occlusion of left circumflex artery (LCx). (**B**) Left anterior oblique caudal view showing chronic 100% occlusion of right coronary artery (RCA). LM, left main coronary artery.

A 76-year-old woman with a history of hypertension, obesity, and asthma presented with 4 days of progressive dyspnea and mild substernal chest discomfort. At presentation, she manifested signs of cardiogenic shock: she was pale, diaphoretic, and hypotensive (80/50 mm Hg, mean arterial pressure 60 mm Hg). Her laboratory studies revealed markedly elevated troponin of 22 ng/mL (indicating substantial myocardial injury) and lactate of 9 mmol/L, signifying profound tissue hypoperfusion. Physical examination found prominent jugular venous distension and bibasilar crackles. Chest radiography demonstrated classic features of pulmonary edema with bilateral pleural effusions. Electrocardiography revealed Q waves and minor ST-segment elevation (<1 mm) in the inferior leads, supporting the diagnosis of a subacute inferior MI. Bedside transthoracic echocardiography identified a left-to-right shunt suggestive of VSR. Emergent coronary angiography confirmed a 100% thrombotic occlusion of the left circumflex artery (Figure 1A) and a chronic right coronary artery occlusion with collaterals (Figure 1B). Right heart catheterization further supported the diagnosis, demonstrating a step-up in oxygen saturation from the right atrium (45%) to the right ventricle (70%), diagnostic for VSR.

**Figure 2:**
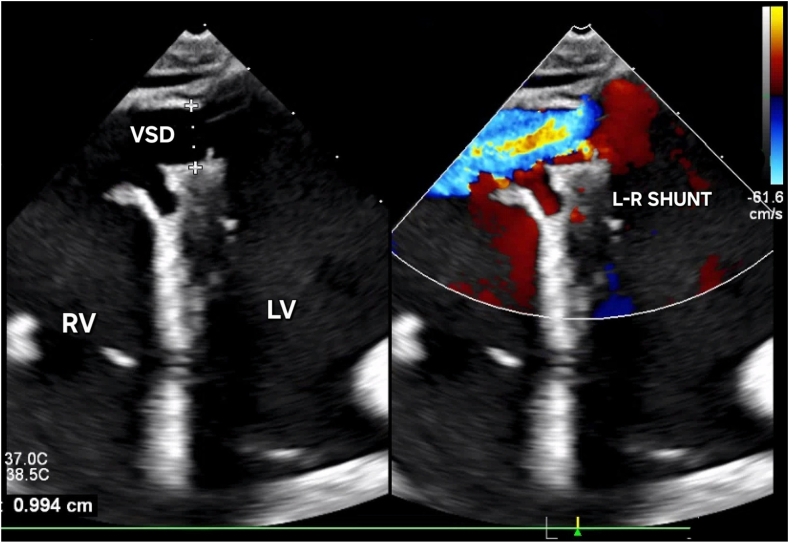
**Transthoracic echocardiogram (on presentation) showing 1-cm VSD with left-to-right shunt.** L-R shunt, left-to-right shunt; LV, left ventricle; RV, right ventricle; VSD, ventricular septal defect.

**Figure 3:**
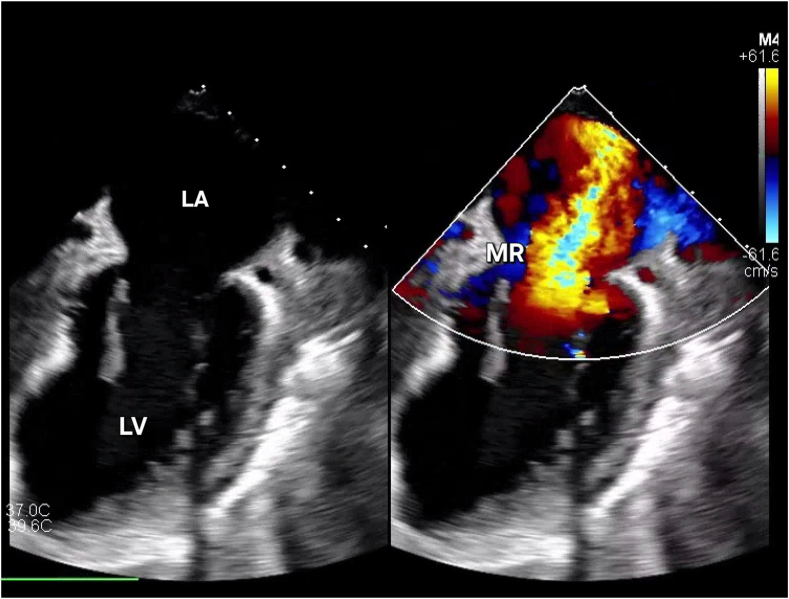
**Transthoracic echocardiogram (on presentation) showing severe MR.** LA, left Atrium; LV, left ventricle; MR, mitral regurgitation.

Due to the patient’s hemodynamic instability, an intra-aortic balloon pump (IABP) was placed to support circulation in conjunction with vasopressors and mechanical ventilation. Given the chronic occlusion and established collaterals to the right coronary artery, revascularization of was not pursued. Serial lactate measurements subsequently normalized, reflecting restoration of critical perfusion. Subsequent echocardiographic and hemodynamic evaluations painted a picture of the mechanical sequelae following the subacute MI. Transthoracic echocardiography quantified a left ventricular ejection fraction of 30%, with global hypokinesis, akinesis of the inferior wall, and right ventricular involvement. Crucially, it detailed an irregularly shaped 1-cm ventricular septal defect (VSD) in the mid-inferoseptum with a prominent left-to-right shunt (Figure 2; Supplemental Video 1), as well as evidence of intramyocardial dissection. A partially torn papillary muscle was also found resulting in severe acute MR (Figure 3; Supplemental Video 1).

However, the hospital course was complicated by new-onset atrial fibrillation with a rapid ventricular response and recurrent ventilator-associated pneumonia, requiring prolonged intensive care unit admission and antimicrobial therapy. IABP support was maintained for 25 days to allow for both stabilization of the septal defect via tissue scarring and control of ongoing infection. Despite partial improvement, the cumulative burden of comorbidities, frailty, and infectious risk precluded open surgical repair of the VSD and MR. Although surgery remains the gold standard for definitive management of postinfarct VSR and MR, operative mortality is often prohibitive in high-risk, debilitated patients. In our case, the multidisciplinary heart team presented the family with 2 palliative, less invasive options: percutaneous VSD closure with mitral valvuloplasty (specifically edge-to-edge repair using MitraClip) or comfort-focused care. As the patient was previously independently functioning in all activities of daily living (including instrumental activities of daily living), the family opted for percutaneous intervention.

**Figure 4:**
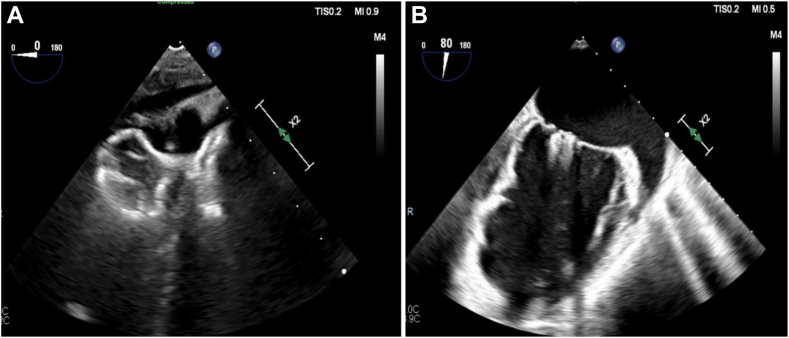
**Transesophageal echocardiogram (after repair).** (**A**) Amplatzer occluder device occluding ventricular septal rupture. (**B**) Two MitraClips for mitral valve repair.

Under combined fluoroscopic and echocardiographic guidance, femoral venous and arterial access was obtained. A retrograde arterial approach allowed wire passage from the left ventricle through the septal defect into the pulmonary artery, where it was snared and externalized via the right femoral vein. This arteriovenous “rail” enabled the deployment of a 16 mm × 10 mm Amplatzer VSD occluder device (Abbott) through a 10F sheath across the septum (Figure 4A; Supplemental Video 1). After proper placement, it was opened on the left ventricular side and pulled back to nest at the VSD. Post-occlusion shunt assessment revealed near complete resolution of the septal defect.

Attention then turned to the mitral valve. Using right femoral venous access, a MitraClip G4 XTW device (Abbott) was advanced transseptally into the left atrium. Using echocardiographic guidance, the clip was positioned below the mitral valve, the leaflets were grabbed, and the clip was closed. The MR improved significantly, but there was residual moderate MR; hence, a G4 NTW clip was placed slightly more medial than the initial clip (Figure 4B). Echocardiographic guidance during transcatheter edge-to-edge repair confirmed improved leaflet coaptation. Hemodynamic measurements showed mild remaining mitral inflow gradient and reversal of pulmonary vein flow, confirming successful reduction in MR severity.

The total radiation dose for the procedures was 6853 cGy·cm^2^, and the cumulative procedure time was 5 hours. Afterward, the IABP was discontinued, but prolonged ventilatory weaning required tracheostomy. After further management of urinary tract infection and ventilator-associated pneumonia during the hospital stay, the patient was discharged to a skilled nursing facility after an extended 2-month hospitalization.

## Discussion

Post-MI mechanical complications, though increasingly rare in the era of early revascularization, continue to pose significant risks to patient survival. VSR exemplifies the severity of these complications by inducing left-to-right shunting, rapid hemodynamic deterioration, and cardiogenic shock. The accompanying tissue friability undermines the success of surgical intervention. When VSR coincides with additional complications, such as PMR, the clinical scenario becomes exceptionally challenging. The optimal timing of surgical repair for post-MI VSR is a subject of ongoing debate, particularly for patients with concomitant mechanical complications, and the procedure must be individualized, factoring in patient's age, comorbidities, shock severity, anticoagulation-related risks, and overall goals of care. Concomitant coronary intervention might be suggested as indicated, and temporary mechanical support can be used to help stabilize ischemic myocardium, which was considered in presented case as well.^3^ Early surgical repair carries a 30% to 50% risk of death; however, mortality rates vary depending on the location of the VSD, with posterior VSD having a 20% and anterior VSD a 45% mortality rate.^10^

In this case, the patient had a late presentation of STEMI and was admitted with cardiogenic shock and respiratory compromise. On presentation, she was diagnosed with combined post-MI PMR and VSD. According to current American Heart Association statements, delayed surgical intervention should be favored when feasible in patients with stable hemodynamic and respiratory parameters, as this approach allows for maturation of infarct borders—including scar formation that enhances suture holding and reduces operative bleeding. Traditionally, open-heart surgery has been the standard recommendation and the reported mortality rate for VSD repair within 1 week is the highest in the modern era of cardiac surgery.^6^ Higher chances of survival are seen in delayed repair, which can be attributed to 2 reasons. First, hearts that are severely damaged often do not survive until the time of repair. Second, the surgery is more effective when the VSD edges have become fibrotic, allowing for easier suturing.^10^

With regard to PMR, emergent mitral valve replacement remains the treatment of choice. For patients who are not candidates for surgical intervention, alternative therapies include transcatheter edge-to-edge repair, medical management as a bridge to valve replacement, and temporary mechanical support as a bridge to long-term ventricular assist device implantation or heart transplantation.

This case illuminates several pivotal themes in the management of post-MI mechanical complications. First, it exemplifies the trend toward patient-tailored intervention, with careful balancing of the risks and timing of surgery against the evolving potential of percutaneous therapies. For high-risk patients—particularly those with frailty, active infection, or prohibitive surgical risk—less invasive approaches may not only represent the safest alternative but also permit simultaneous resolution of multiple anatomic lesions within a single procedure. Our case also highlights a crucial area of research, emphasizing that the percutaneous approach should be considered a viable option for debilitated patients who are not candidates for open-heart surgery. Additionally, it demonstrates the potential to address multiple complications in a single procedure.

## Conclusion

Post-MI VSR and PMR, while increasingly uncommon, remain lethal when they occur, particularly in combination. Although surgical repair has traditionally offered the best outcomes, select patients may benefit from percutaneous closure strategies, especially when comorbidities or persistent instability preclude open-heart surgery. Early recognition, multidisciplinary planning, mechanical support, and evolving transcatheter technologies together offer hope for improved survival and recovery in this challenging patient population. As highlighted by this case, individualized care and continued innovation are imperative for advancing the management of postinfarction mechanical complications.
